# Repeated SARS-CoV-2 Positivity: Analysis of 123 Cases

**DOI:** 10.3390/v13030512

**Published:** 2021-03-19

**Authors:** Szilárd Váncsa, Fanni Dembrovszky, Nelli Farkas, Lajos Szakó, Brigitta Teutsch, Stefania Bunduc, Rita Nagy, Andrea Párniczky, Bálint Erőss, Zoltán Péterfi, Péter Hegyi

**Affiliations:** 1 Institute for Translational Medicine, Medical School, University of Pécs, 7624 Pécs, Hungary; vancsaszilard@gmail.com (S.V.); dembrovszky.f@gmail.com (F.D.); farkas.nelli@gmail.com (N.F.); szaklaj@gmail.com (L.S.); teutschbrigitta@gmail.com (B.T.); stfnbndc@gmail.com (S.B.); nagyrita003@gmail.com (R.N.); andrea.parniczky@gmail.com (A.P.); dr.eross.balint@gmail.com (B.E.); 2 János Szentágothai Research Center, University of Pécs, 7624 Pécs, Hungary; 3 Institute of Bioanalysis, Medical School, University of Pécs, 7624 Pécs, Hungary; 4 Fundeni Clinical Institute, Gastroenterology, Hepatology and Liver Transplant Department, 022328 Bucharest, Romania; 5 Doctoral School, Carol Davila University of Medicine and Pharmacy, 050474 Bucharest, Romania; 6 Heim Pál National Pediatric Institute, 1089 Budapest, Hungary; 7 Doctoral School of Theoretical Medicine, Faculty of Medicine, University of Szeged, 6720 Szeged, Hungary; 8 Division of Infectious Diseases, First Department of Medicine, Medical School, University of Pécs, 7624 Pécs, Hungary

**Keywords:** SARS-CoV-2, COVID-19, new coronavirus, polymerase chain reaction, positive, repeated, case reports, systematic review

## Abstract

Repeated positivity and reinfection with severe acute respiratory syndrome coronavirus 2 (SARS-COV-2) is a significant concern. Our study aimed to evaluate the clinical significance of repeatedly positive testing after coronavirus disease 2019 (COVID-19) recovery. We performed a systematic literature search following the Preferred Reporting Items for Systematic Reviews and Meta-Analyses (PRISMA) guideline. With available individual patient data reporting on repeatedly SARS-CoV-2 positive (RSP) patients, case reports, and case series were included in this analysis. We performed a descriptive analysis of baseline characteristics of repeatedly positive cases. We assessed the cases according to the length of their polymerase chain reaction (PCR) negative interval between the two episodes. Risk factors for the severity of second episodes were evaluated. Overall, we included 123 patients with repeated positivity from 56 publications, with a mean repeated positivity length of 47.8 ± 29.9 days. Younger patients were predominant in the delayed (>90 days) recurrent positive group. Furthermore, comparing patients with RSP intervals of below 60 and above 60 days, we found that a more severe disease course can be expected if the repeated positivity interval is shorter. Severe and critical disease courses might predict future repeatedly positive severe and critical COVID-19 episodes. In conclusion, our results show that the second episode of SARS-CoV-2 positivity is more severe if it happens within 60 days after the first positive PCR. On the other hand, the second episode’s severity correlates with the first.

## 1. Introduction

Coronavirus disease 2019 (COVID-19) pandemic affected more than 100 million patients and caused more than 2 million deaths globally when writing this report, being the most challenging healthcare crisis during the past century [1]. Active immunization for disease prevention seems to be the most feasible solution to curb the pandemic’s medical, economic, and social impact [2]. In light of this, the emerging data on repeatedly positive reverse-transcription polymerase chain reaction (PCR) samples for severe acute respiratory syndrome coronavirus 2 (SARS-COV-2) after initial recovery from COVID-19 are of major concern [3].

The reported incidence of repeatedly positive cases among the patients who recovered from COVID-19 ranges from 2.4 to 69.2%, and the reasons for repeated positivity are unclear [3]. Multiple mechanisms have been considered, including reinfection, disease relapse, prolonged viral shedding, and laboratory or technical errors [4]. Regarding reinfection, the European Center for Disease Prevention and Control recommends whole-genome sequencing to compare the strains responsible for each episode, yet very few studies report sequencing data [5,6,7,8].

Because PCR can be positive for up to 100 days in upper respiratory tract samples, viral viability should be verified by cell culture or viral load quantification to differentiate the shedding of viral ribonucleic acid fragments from actual infection [5]. Reports have been conflicting, some of them describing a milder, others a more severe disease course at the time of second PCR positivity [9,10]. No demographic or clinical risk factors for repeated positivity have been identified, and no infections were reported among the contacts of patients with repeatedly positive tests [11]. Although infection induces the development of neutralizing antibodies in more than 90% of cases, it is unclear if and how long they provide protection [9,12].

Our study aimed to evaluate the clinical significance of repeatedly positive testing after COVID-19 recovery regarding predisposing factors for more severe symptoms and the second episode’s disease course. We also describe repeatedly positive cases’ baseline characteristics and assess them according to the length of their PCR-negative interval between the two episodes.

## 2. Materials and Methods

We performed a systematic literature search according to Preferred Reporting Items for Systematic Reviews and Meta-Analyses (PRISMA) guideline (Table S1) [13]. The review was registered on PROSPERO under the ID number CRD42021228422 (see https://www.crd.york.ac.uk/prospero (accessed on 2 February 2021)) in advance.

We used the following PICO framework: (P): repeatedly SARS-CoV-2 positive (RSP) patients with two positive PCRs separated by a negative PCR test result, (I/C) gender, comorbidities, severity and presenting symptoms, and (O) severity of the second episode, and the time interval between repeated positivity.

### 2.1. Search and Selection

The MEDLINE (via PubMed), Embase, and Cochrane Central Register of Controlled Trials (CENTRAL) databases were searched until 22 November 2020 for relevant case reports, with the following search key: ((“covid 19”) OR (“coronavirus”) OR (“2019 nCoV”) OR (“SARS-cov-2”)) AND ((reinfection) OR (“second episode”) OR (“second infection”)).

With available individual patient data reporting on repeatedly COVID-19-PCR positive patients, case reports, and case series were included in this analysis. Cohort and case-control studies were excluded. A total of two review authors performed the selection by title, abstract, and full text independently. Disagreements were resolved by consensus.

### 2.2. Data Extraction

Relevant data, including the year of publication, name of the first author, age, gender, and existing comorbidities of the patient, and severity, symptoms, imaging, and laboratory findings of first and second episodes of the disease course, were extracted to a pre-defined Excel (Microsoft Corporation, Redmond, Washington, United States) datasheet. A total of two independent review authors performed data extraction and resolved the disagreements by consensus.

We included only nasal swab PCR-confirmed COVID-19 cases in the analysis. COVID-19 was defined as a positive PCR of SARS-nCoV-2.

Severity was assessed based on the guidelines on the Diagnosis and Treatment of COVID-19 issued by the National Health Commission of China [14]. In case of missing data regarding severity, one review author (Z.P.) classified the cases based on the patient’s symptoms following the mentioned guideline. A detailed description of the classification system is included in Table S2.

**Figure 1:**
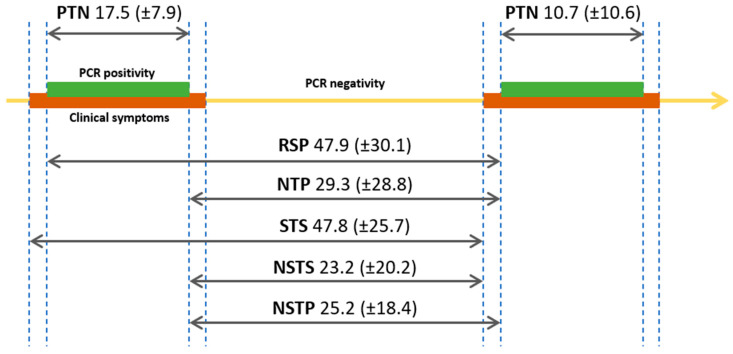
Summary of the analyzed time intervals. Numbers represent mean days with Scheme 2. positivity; STS: symptom to symptom interval.

Repeated positivity was defined as two positive PCRs separated by a negative PCR test result. The RSP interval represents the interval between the two positive PCRs of each episode. Patients were classified based on the length of the RSP interval. Details of the analyzed intervals are presented in Figure 1.

### 2.3. Statistical Analysis

We generated and then analyzed the cohort of the included cases. Descriptive statistics were performed to characterize the RSP population. The association between categorical variables was examined with the Chi-square and Fisher’s exact tests. To assess the differences between groups t-test was applied for normally distributed variables and the Mann–Whitney U test for non-normal data. The Kruskal–Wallis test was used to compare more than two groups, with Bonferroni post-hoc adjustment. For correlation between categorical parameters, linear regression was used. A *p*-value of <0.05 was considered statistically significant. Statistical analyses were performed using International Business Machines Corporation (IBM)-SPSS for Windows 25 software (IBM Corp, Armonk, NY, USA).

A subgroup analysis was performed, according to RSP intervals: (a) 1–30 days, (b) 31–60 days, (c) 61–90 days, or (d) more than 90 days between their two positive PCR positive episodes.

### 2.4. Data Quality

Data quality is detailed in Table S3. Data quality represents the percentage of available data for each parameter in the cohort. Parameters with a data quality under 50% were not included in our analysis.

### 2.5. Assessment of Risk of Bias

To assess the risk of bias of case reports and case series, we applied the Joanna Briggs Institute Critical Appraisal Checklist tools [15]. Then, two independent review authors assessed the risk of bias, and disagreements were resolved by consensus.

## 3. Results

The selection process is detailed in Figure S1. We identified 1612 records in three databases for evaluation. After the removal of duplicates, screening, and selection,198 full texts were assessed. Altogether, 56 articles fulfilled the eligibility criteria and were included in our systematic review and analysis.

### 3.1. Characteristics of the Included Studies

The main characteristics of the included studies are summarized in Tables S4 and S5. Of the 56 included studies from 18 countries, nine were case series describing 65 RSP patients, and 47 were case reports with 58 patients. In total, 123 patients with RSP were assessed in our analysis. Most of the studies were published from China (*n* = 22), Italy (*n* = 7), and the USA (*n* = 5).

Overall, 56% of the data required for our analyses were provided. Data were almost complete on age, gender, the severity, and symptoms of COVID-19. We could not analyze laboratory parameters due to limited data. Data were 100% complete for the interval between the two COVID-19 episodes.

### 3.2. Characteristics of the Cohort

**Figure 2:**
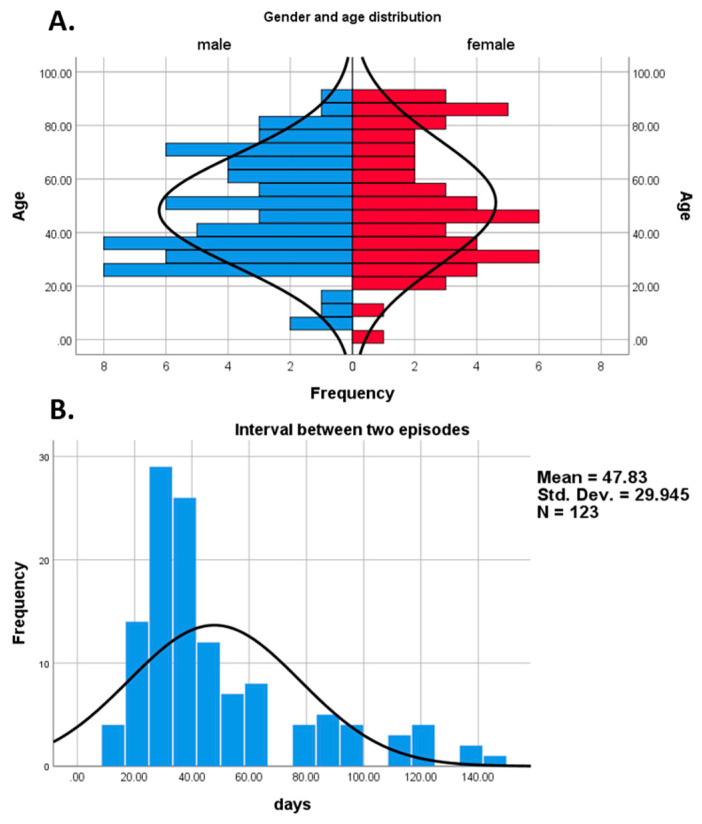
Age distribution of patients with repeatedly SARS-CoV-2 positivity (**A**), and the histogram of repeated positivity lengths (**B**).

The main characteristics of the analyzed cohort are shown in Table 1. In our cohort, the mean age was 49.7 (SD 21.9, range 1–93), and 45.1% of the patients were female (*n* = 55/123). Age distribution based on gender is presented in Figure 2A. The most frequent comorbidities were hypertension (*n* = 22/82, 26.8%) and diabetes mellitus (*n* = 19/82, 23.2%). Overall, 66.3% (*n* = 57/86) of the patients had at least one comorbidity. During the first and second positive episodes, most of the patients were hospitalized (*n* = 95/119, 72.2% vs. *n* = 87/111, 70.7%, respectively).

Despite the high hospitalization rate, most of the cases were mild and moderate (*n* = 86/109, 78.9% and *n* = 81/97, 82.7%, respectively), and only few patients developed a critical condition (*n* = 7/109, 6.4% and *n* = 9/97, 9.3%, respectively) during the first and second episodes. The most frequent symptoms were fever (*n* = 76/108, 70.4%), cough (*n* = 67/108, 62%), and dyspnea (*n* = 28/105, 26.7%) during the first episode, compared to the second episode, where these symptoms were present in a smaller proportion (*n* = 30/100, 30%; *n* = 29/97, 29.9%; *n* = 27/100, 27%, respectively). Pneumonia was present in similar proportion during the first and second episodes (*n* = 46/62, 74.2% vs. *n* = 51/61, 83.6%); however, the data quality was borderline low for this parameter in the two episodes (50.41 and 49.59%, respectively). Immunoglobulin G was positive in 86.1% (*n* = 31/36) and 94.2% (*n* = 49/52) of the patients after the first and second episode, respectively.

Asymptomatic patients were present in 6.12% (*n* = 6/98) and 41.94% (*n* = 39/93) during the first and second episodes, respectively. A total of three cases presented symptoms between the two episodes, although they presented with negative COVID-19 PCR test results. Overall, out of the 123 patients only three deaths occurred during the second PCR positive episode, and three patients were in the hospital on reporting.

### 3.3. Length of Positivity and Intervals between Episodes

The first episode’s duration was significantly longer than the second episode’s (17.53 ± 7.86 vs. 10.71 ± 10.63 days, respectively; *p* = 0.001). The length of RSP for each patient was calculated, the mean interval being 47.9 ± 30.1 days (Figure 2B). The mean length of the rest of the analyzed intervals is presented in Figure 1 and Figure S2. The mean and median length for the negative to positive (NTP) interval was 29.3 (±28.8 SD) days, and 17 (10.75-37.25 Q1-Q3) days, respectively.

**Figure 3:**
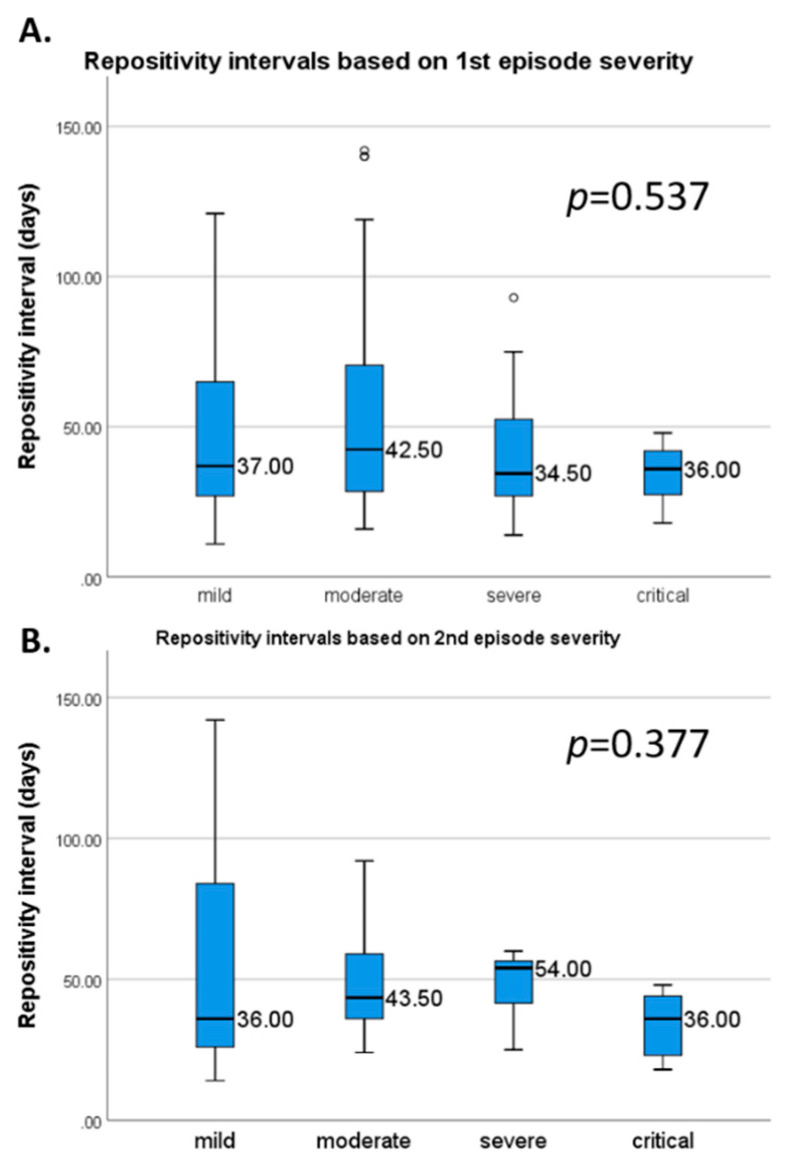
Median repeated positivity lengths based on the first (**A**) and second episode severity (**B**). Error bars represent 1.5 times the interquartile range.

We found that younger patients might have a second episode later than older patients, although without statistical significance (R = 0.064, *p* = 0.486, Figure S3). Median RSP intervals based on the first- and second-episodes severity are presented in Figure 3. Mean RSP intervals for each parameter are shown in Table 2. Only the hospitalization during the first episode (44.2 ± 29.3 vs. 64.6 ± 29.8 days, respectively; *p* = 0.001) and the presence of pneumonia on imaging examinations (35.3 ± 12.9 vs. 70.8 ± 40.9 days, respectively; *p* = 0.012) during the second episode was associated with a shorter RSP interval. On the other hand, only the presence of headache during the first episode was associated with a longer mean RSP interval (63.8 ± 38.8 vs. 47.6 ± 30.1 days, respectively; *p* = 0.042).

### 3.4. Comparing Patients with below and above 60 Days of RSP

Based on Figure 2B, we compared patients with less than 60 days between RSP episodes with those with more than 60 days. A summary of the findings is included in Table 1. The mean RSP interval in the below 60 days group was 34.1 ± 11.4 days (*n* = 96), while in the above 60 days group was 97 ± 24 days (*n* = 27), with a significant difference between the groups (*p* = 0.001). The two groups were not different regarding age (51 ± 22.3 vs. 45.2 ± 20.2 years; *p* = 0.228) and gender (female *n* = 42/96, 43.8% vs. *n* = 13/26, 50%; *p* = 0.570). Similarly, most of the listed parameters were comparable between the two groups.

On the other hand, hypertension was more frequent in the below 60 days group (*n* = 20/61, 32.8% vs. *n* = 2/21, 9.5%; *p* = 0.038). During the first episode, hospitalization (*n* = 79/119, 85.9 vs. *n* = 16/27, 59.3%; *p* = 0.002) and gastrointestinal symptoms (*n* = 20/82, 24.4% vs. *n* = 0/27, 0%; *p* = 0.005) were more frequent in the below 60 days group; however, the length of the episode was longer in the above 60 days group (16.5 ± 6.9 vs. 21.1 ± 10 days, *p* = 0.016).

During the second episode, hospitalization was similarly more frequent in the below 60 days group (*n* = 73/88, 83% vs. *n* = 14/23, 60.9%, *p* = 0.043). Furthermore, pneumonia (*n* = 49/53, 92.5% vs *n* = 2/8, 25%, *p* = 0.001) and dyspnea (*n* = 24/74, 32.4% vs *n* = 3/26, 11.5%, *p* = 0.039) were also more frequent in the below 60 days group. We found a significant difference in severity, severe (*n* = 7/71, 9.9% vs. *n* = 0/26, 0%), and critical (*n* = 9/71, 12.7% vs. *n* = 0/26, 0%; *p* = 0.039) cases being more frequent in the below 60 days group.

### 3.5. Comparison Based on Intervals

**Figure 4:**
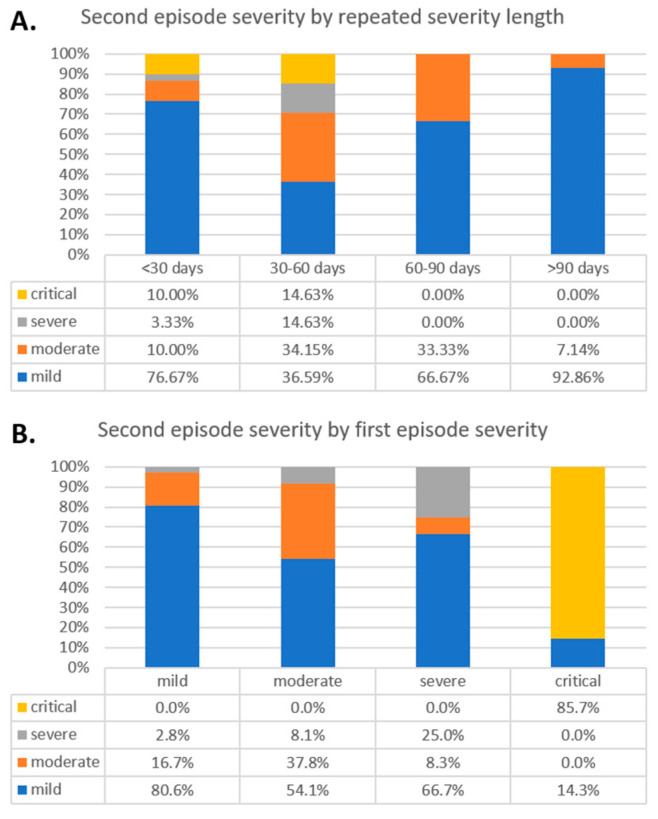
Severity based on repeated positivity intervals (**A**), and second episode’s severity based on the first episode severities (**B**).

We divided RSP patients into four groups (below 30, 30–60, 60–90, and above 90 days), based on the length of the RSP intervals. The summary of findings is included in Table 3. The severity distributions based on the selected intervals are presented in Figure 4A. The critical and severe second episodes were more frequent in the below 30- and 30–60-day interval (*p* = 0.005), compared to the above 60 groups. Similarly, the difference was present regarding the first episode’s severity; however, the difference was non-significant (*p* = 0.797).

Among the groups, the mean age was the highest (56.4 ± 20.5 years) in the 30 to 60 days group, and the lowest in the above 90 days group (36.6 ± 16.1 years), with a significant difference between groups (*p* = 0.001). Regarding comorbidities, cancer was also more frequent in the 60 to 90 days group (22.2%, *p* = 0.015). During the first episode, hospitalization was higher in the below 30 and 30–60-days group (95 and 78.8%, respectively; *p* = 0.005). Gastrointestinal symptoms were present only in the below 30 and 30–60-days group (*p* = 0.045), with no other significant differences regarding symptoms. Lastly, during the second episode, pneumonia, and dyspnea were most frequent in the between 30–60-days group (96.6 and 44.2%, respectively).

### 3.6. The Severity of the First and Second Episodes

According to the first episode’s severity, the severity of the second episode is presented in Figure 4B. After mild and moderate first episodes, most of the second episodes were similarly mild or moderate (97.3 and 91.9%, respectively). During the second episode, severe cases were more common if the first episode was severe (25%). Lastly, critical cases tended to stay critical (85.7%). We did not find significant difference in the analyzed intervals based on first- and second-episode severity.

**Figure 5:**
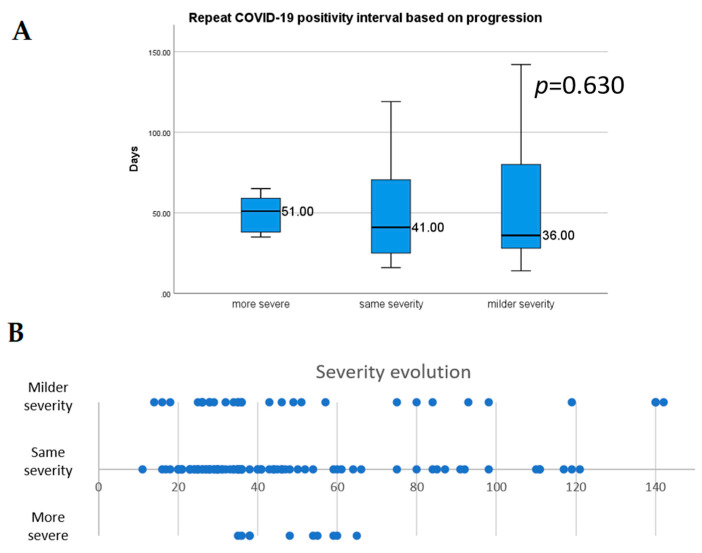
Median repeated positivity length based on progression (**A**), and distribution of the progression of the second episode (**B**). Error bars represent 1.5 times the interquartile range.

In Figure 5, the evolution of severity is presented. The mean RSP interval was similar when comparing progression direction (*p* = 0.630); in the case of a worse severity during the second episode compared to the first episode, the mean RSP interval was 48.8 ± 11.3 days, for a similar severity, it was 50.7 ± 30.8 days, and in the case of a less severe course the mean was 55.4 ± 39 days.

Lastly, the mean and median RSP interval for asymptomatic patients are summarized in Table S6. RSP interval was higher in asymptomatic patients.

### 3.7. Risk of Bias Assessment

The summary of the risk of bias assessment is shown in Tables S8 and S9. We waived the scoring of the statistical analysis domain in case series since it was not a factor of interest.

## 4. Discussion

Our analysis aimed to systematically review case reports and case series, with individual patient data reporting on repeated COVID-19 positivity. We found that repeatedly positive patients did not differ from patients who had only one episode of COVID-19. In a meta-analysis of more than 3000 patients with a single episode of COVID-19, patients were predominantly male, similarly to our findings, while the same symptoms dominated in our analyzed cohort (fever, cough, and dyspnea) [16]. We generally see a lower prevalence of the analyzed symptoms during the second episode, which correlates with the higher proportion of asymptomatic or mild cases. The proportion of pneumonia during each episode was high, although data was present only in 50% of the cases.

Based on the correlation between the age and RSP length, younger patients might have a second episode later than older patients. Similarly, in our cohort younger patients were predominant in the delayed (>90 days) repeated positivity group, which are more likely to represent actual reinfections [17]. Unfortunately, serology and viral viability data were scarce in our cohort. Nevertheless, knowing that milder cases are more prevalent among youth, our data suggest that younger patients might be more prone to reinfection because of a lack of efficacious active immunization after a less severe first episode of disease [18,19].

Shorter RSP interval was associated with increased number of pneumonia and longer hospital stay during the second episode. Furthermore, comparing patients with RSP intervals of below 60 and above 60 days, we found that a more severe disease course can be expected if the RSP interval is shorter. These cases can probably be explained by a continued and aggravated disease course with false-negative PCR results midpoint in their illness rather than genuine reinfection [17]. Furthermore, the higher number of pneumonias in the case of below 30 days RSP group also strengthens this theory. Lastly, in our cohort the severe and critical disease courses might be predictive of future severe and critical episodes.

To our knowledge, this is the first systematic review and meta-analysis of case reports and case series on repeatedly SARS-CoV-2 positive patients. Previous studies aimed to assess the question of repeated positivity, with different results. One of these focused on the relapse of the disease, which is one of the potential causes of repeated positivity. It is noteworthy that none of the patients included in this study were asymptomatic at the time of relapse, which could act well to differentiate between reinfection and relapse [20]. In accordance, another study of 182 patients reported that repeated positivity during isolation after the initial episode of infection is rarely associated with the recurrence of disease if symptoms are absent [21]. Another meta-analysis concluded that to diagnose reinfection the RSP interval needs to be above 90 days and the patients must have symptoms [22]. Similarly, both the Center for disease Control and Prevention and the Health Protection Surveillance Center recommend an interval of at least 90 days to consider for the possibility of a reinfection [23,24].

Although multiple reports have described recurrent positivity after an initial episode, most of them did not differentiate between reinfection, recurrent positivity, relapse, or false positive tests. In our analysis we tried to assess the clinical importance of repeated positivity, but we cannot ignore the importance of reinfection. In our analyzed cohort we found a short NTP interval, which can correlate with long lasting resolution in some of the cases, rather than reinfection.

After more than one year since the first reported case of COVID-19, a consensus regarding reinfection by SARS-CoV-2 is still lacking. To diagnose reinfection, it requires the evidence of a new infection by a phylogenetically distinct form of the SARS-CoV-2 virus after the elimination of the previous one [7,23].

Out of the 56 articles included in our analysis, only eight described different strains during the two COVID-19 episodes (see Table S10) [6,7,25,26,27,28,29].

On the other hand, long shedding might be the most common background of repeated positivity. In a study of 38 long-term SARS-CoV-2 carrier patients, the median carrying history was 92 days after the onset of COVID-19, and the longest reported period was 118 days. During these periods, negative-positive PCR result fluctuation were observed [30]. Another study reported on 99 patients with a positive SARS-CoV-2 PCR after 4 weeks from the first positive viral test [31].

In our analyzed cohort, a high percentage of patients were asymptomatic during the second episode. Data in the literature regarding cycle threshold (Ct) value in asymptomatic patients is contradictory. There are studies that show Ct values are higher for asymptomatic patients while other show no difference by comparison with the symptomatic cases [32,33]. On the other hand, Ct value could be used as a surrogate marker to assess viral viability, because viral culture, is not widely available and requires high level biosafety [34]. The CDC recommends Ct value under 33 for cases diagnosed only by one positive PCR [23], while other authors suggest two positive tests with Ct < 35 as criteria for reinfection [17].

### 4.1. Strengths and Limitations

One of the strengths of our analysis is the preregistered protocol, which prevents publication bias. To our knowledge, this analysis is the most comprehensive work, also including individual patient data.

However, our work has multiple limitations. Most of the included case series and case reports are retrospective. The case series did not follow the Case Report (CARE) guidelines when reporting individual cases. Most of the studies lacked follow-up of the infected patients. To confirm a reinfection, one should assess the strain of the virus, which was not done in most of the articles. The case series themselves carry the limitation of publication bias, general overinterpretation, furthermore these are not representative for the whole COVID-19 population. In the case of case reports, mostly rare and unusual cases are reported, while being outcome oriented.

### 4.2. Implication for Research

We strongly emphasize the conduction of follow-up studies, with PCR testing after the convalescence of the disease. Cases with high Ct values should be considered with caution and ideally viral viability should be assessed if available to confirm reinfection. Animal model studies are also needed to assess the possibility of reinfection with the same strain. Based on these findings we might be able to create a classification, which can distinguish between false positivity, relapse, and reinfection.

### 4.3. Implication for Practice

From a practical point of view critical evaluation of a repeated positivity is suggested. One should assess whether it is associated with symptoms, how long the time interval between the two episodes is and whether the cycle threshold of the second PCR is too high.

## 5. Conclusions

In summary, our results show that the second episode of SARS-CoV-2 positivity is more severe if it happens within 60 days after the first positive PCR. On the other hand, the second episode severity correlates with the first infection.

## Figures and Tables

**Table 1:** Baseline characteristics of the overall analyzed population and comparison of patients with repeated positivity length under 60 with patients above 60 days.

Parameter	Overall *n*/N (% Total)	≤60 Days *n*/N (% Total)	>60 Days *n*/N (% Total)	*p*-Value
Total number N	123	96	27	
Female	55/122 (45.1)	42/96 (43.8)	13/26 (50)	0.570
Age (mean, SD, range)	49.7 (21.9)	51 (22.3)	45.2 (20.2)	0.228
Mean repeated positivity interval (SD)	47.9 (30.1)	34.1 (11.4)	97 (24)	0.001
Comorbidities				
Hypertension	22/82 (26.8)	20/ 61 (32.8)	2/ 21 (9.5)	0.038
Chronic heart disease	14/84 (16.7	11/ 63 (17.5)	3/ 21 (14.3)	0.735
Arrhythmia	11/84 (13.1)	8/ 63 (12.7)	3/ 21 (14.3)	0.852
T2DM	19/82 (23.2)	15/ 61 (24.6)	4/ 21 (19.1)	0.604
COPD	6/82 (7.3)	4/ 61 (6.6)	2/ 21 (9.5)	0.653
Chronic kidney disease	5/82 (6.1)	5/ 61 (8.2)	0/ 21 (0)	0.176
Chronic liver disease	2/82 (2.4)	1/ 61 (1.6)	1/ 21 (4.8)	0.424
Immunosuppression	13/82 (15.9)	9/ 63 (14.3)	4/ 19 (21.1)	0.479
Cancer	3/84 (3.6)	1/ 63 (1.6)	2/ 21 (9.5)	0.090
Other	31/78(39.7)	21/ 58 (36.2)	10/ 20 (50)	0.277
**First episode**				
Mean days of positivity (Mean, SD)	17.5 (7.9)	16.5 (6.9)	21.1 (10)	0.016
Mild COVID-19	46/109 (42.2)	32/ 82 (39)	14/ 27 (51.9)	0.208
Moderate COVID-19	40/109 (36.7)	29/ 82 (35.4)	11/ 27 (40.7)	
Severe COVID-19	16/109 (14.7)	14/ 82 (17.1)	2/ 27 (7.4)	
Critical COVID-19	7/109 (6.4)	7/ 82 (8.5)	0/ 27 (0)	
Hospitalization	95/119 (77.2)	79/ 92 (85.9)	16/ 27 (59.3)	0.002
Pneumonia	46/62 (74.2)	39/51 (76.5)	7/11 (63.3)	0.452
Fever	76/108 (70.4)	60/ 81 (74.1)	16/ 27 (59.3)	0.144
Cough	67/108 (62)	52/ 81 (64.2)	15/ 27 (55.6)	0.423
Dyspnea	28/105 (26.7)	20/ 78 (25.6)	8/ 27 (29.6)	0.686
Arthromyalgia	20/108 (18.5)	15/ 81 (18.5)	5/ 27 (18.5)	1.000
Headache	13/108 (12)	9/ 81 (11.1)	4/ 27 (14.8)	0.609
General cold symptoms	19/108 (17.6)	15/ 81 (18.5)	4/ 27 (14.8)	0.662
Asthenia	14/109 (12.8)	11/ 82 (13.4)	3/ 27 (11.1)	0.756
Gastrointestinal symptoms	20/109 (18.3)	20/ 82 (24.4)	0/ 27 (0)	0.005
**Second episode**				
Mean days of positivity (Mean, SD)	10.7 (10.6)	10.6 (10.7)	10.9 (10.9)	0.928
Mild COVID-19	59/97 (60.8)	38/ 71 (53.5)	21/ 26 (80.8)	0.039
Moderate COVID-19	22/97 (22.7)	17/ 71 (23.9)	5/ 26 (19.2)	
Severe COVID-19	7/97 (7.2)	7/ 71 (9.9)	0/ 26 (0)	
Critical COVID-19	9/97 (9.3)	9/ 71 (12.7)	0/ 26 (0)	
Hospitalization	87/111 (70.7)	73/ 88 (83)	14/ 23 (60.9)	0.043
Pneumonia	51/61 (83.6)	49/53 (92.5)	2/8 (25)	0.001
Fever	30/100 (30)	24/ 74 (32.4)	6/ 26 (23.1)	0.371
Cough	29/97 (29.9)	24/ 71 (33.8)	5/ 26 (19.2)	0.165
Dyspnea	27/100 (27)	24/ 74 (32.4)	3/ 26 (11.5)	0.039
Arthromyalgia	19/99 (19.2)	14/ 73 (19.2)	5/ 26 (19.2)	1.000
Headache	10/100 (10)	7/ 74 (9.5)	3/ 26 (11.5)	0.717
General cold symptoms	16/100 (16)	12/ 74 (16.2)	4/ 26 (15.4)	1.000
Asthenia	8/100 (8)	5/ 74 (6.8)	3/ 26 (11.5)	0.425
Gastrointestinal symptoms	9/100 (9)	8/ 74 (10.8)	1/ 26 (3.8)	0.439

COPD: chronic obstructive pulmonary disease; COVID-19: Coronavirus disease 2019; SD: standard deviation; T2DM: type 2 diabetes mellitus.

**Table 2:** Mean repeated positivity length based on the listed parameters.

Parameter	Number with and without Assessed Parameter	Mean RSP (SD) Parameter Present	Mean RSP (SD) Parameter Absent	*p*-Value
Female	55	47.7 (31.6)	−	0.651
Male	67	46.7 (26.9)	−	
Comorbidities				
Hypertension	22 vs. 60	44.8 (24.8)	55.8 (32.5)	0.379
Chronic heart disease	14 vs. 70	46.8 (20.2)	53.6 (32.3)	0.773
Arrhythmia	11 vs. 73	49.2 (23.3)	53 (31.7)	0.974
T2DM	19 vs. 63	46.2 (22.6)	54.9 (32.8)	0.271
COPD	6 vs. 76	61.8 (39.8)	52.2 (30.3)	0.345
Chronic kidney disease	5 vs. 77	46.6 (9.6)	53.3 (31.7)	0.651
Chronic liver disease	2 vs. 80	58 (38.2)	52.7 (30.9)	0.939
Immunosuppression	13 vs. 69	52.9 (33.7)	51.6 (30.3)	0.899
Cancer	3 vs. 81	72.7 (23.1)	51.7 (30.7)	0.123
Other	31 vs. 47	53.8 (27.3)	52.7 (32.5)	0.434
First episode				
Mild COVID-19	46	51.3 (32.9)	−	0.537
Moderate COVID-19	40	54 (34.7)	−	
Severe COVID-19	16	41.1 (20.6)	−	
Critical COVID-19	7	34.4 (11.4)	−	
Hospitalization	95 vs. 24	44.2 (29.3)	64.6 (29.8)	0.001
Pneumonia	46 vs. 16	42.8 (28.2)	42.2 (28.9)	0.342
Fever	76 vs. 32	48.1 (31.4)	53.1 (32.1)	0.638
Cough	67 vs. 41	47.7 (32.6)	52.6 (30)	0.209
Dyspnea	28 vs. 77	48.4 (25.6)	50.7 (34)	0.873
Arthromyalgia	20 vs. 88	46.7 (20.8)	50.2 (33.6)	0.687
Headache	13 vs. 95	63.8 (38.8)	47.6 (30.1)	0.042
General cold symptoms	19 vs. 89	53.6 (35.9)	48.7 (30.7)	0.475
Asthenia	14 vs. 95	46.2 (28)	49.8 (32.1)	0.942
Gastrointestinal symptoms	20 vs. 89	33.8 (10.5)	52.8 (33.6)	0.061
Second episode				
Mild COVID-19	59	55.1 (37.8)	−	0.377
Moderate COVID-19	22	48.5 (18.7)	−	
Severe COVID-19	7	47.9 (13.1)	−	
Critical COVID-19	9	33.9 (11.4)	−	
Hospitalization	87 vs. 24	44 (27.9)	56.4 (37)	0.148
Pneumonia	51 vs. 10	35.3 (12.9)	70.8 (41)	0.012
Fever	30 vs. 70	48.9 (26.1)	51.9 (33)	0.798
Cough	29 vs. 68	47.2 (26)	53.5 (33.3)	0.750
Dyspnea	27 vs. 73	48.3 (25)	52 (33.1)	0.661
Arthromyalgia	19 vs. 80	53 (28.2)	50.6 (32)	0.344
Headache	10 vs. 90	60.8 (35.3)	49.9 (30.5)	0.186
General cold symptoms	16 vs. 84	58.8 (36.6)	49.5 (29.9)	0.220
Asthenia	8 vs. 92	65.3 (38.1)	49.8 (30.3)	0.155
Gastrointestinal symptoms	9 vs. 91	39.1 (15.2)	52.2 (32)	0.511

COPD: chronic obstructive pulmonary disease; COVID-19: Coronavirus disease 2019; SD: standard deviation; RSP: repeatedly severe acute respiratory syndrome coronavirus 2 positivity; T2DM: type 2 diabetes mellitus. Minus sign means no data was available

**Table 3:** Characteristics of the analyzed patients based on the repeated positivity intervals.

Parameter	≤30 Days	31 to 60 Days	61 to 90 Days	>90 Days	*p*-Value
Total number	42	54	12	15	
Female	22/42 (52.4)	20/54 (37)	5/12 (41.7)	8/14 (57.1)	0.363
Age (mean, SD, range)	43.9 (22.9)	56.4 (20.5)	55.9 (20.3)	36.6 (16.1)	0.001
Mean repeated positivity interval (SD)	24.1 (4.9)	41.9 (8.5)	75.5 (9.3)	114.1 (17)	0.001
Comorbidities					
Hypertension	31.25%	33.33%	11.11%	8.33%	0.118
Chronic heart disease	17.65%	17.39%	33.33%	0.00%	0.238
Arrhythmia	11.76%	13.04%	33.33%	0.00%	0.166
T2DM	25.00%	24.44%	33.33%	8.33%	0.557
COPD	0.00%	8.89%	11.11%	8.33%	0.651
Chronic kidney disease	0.00%	11.11%	0.00%	0.00%	0.223
Chronic liver disease	0.00%	2.22%	11.11%	0.00%	0.314
Immunosuppression	17.65%	13.04%	42.86%	8.33%	0.199
Cancer	0.00%	2.17%	22.22%	0.00%	0.015
Other	37.50%	35.71%	66.67%	36.36%	0.377
**First episode**					
Mean days of positivity (Mean, SD)	13.5 (5.3)	19.3 (7)	23.3 (9.8)	19.2 (10.2)	0.001
Mild COVID-19	40.00%	38.30%	50.00%	53.33%	0.797
Moderate COVID-19	37.14%	34.04%	41.67%	40.00%	
Severe COVID-19	17.14%	17.02%	8.33%	6.67%	
Critical COVID-19	5.71%	10.64%	0.00%	0.00%	
Hospitalization	95.0%	78.8%	58.3%	60.0%	0.005
Pneumonia	63.0%	91.7%	75.0%	57.1%	0.452
Fever	69.44%	77.78%	58.33%	60.00%	0.422
Cough	69.44%	60.00%	50.00%	60.00%	0.641
Dyspnea	22.86%	27.91%	41.67%	20.00%	0.569
Arthromyalgia	16.67%	20.00%	33.33%	6.67%	0.349
Headache	2.78%	17.78%	8.33%	20.00%	0.147
General cold symptoms	16.67%	20.00%	16.67%	13.33%	0.941
Asthenia	10.81%	15.56%	8.33%	13.33%	0.884
Gastrointestinal symptoms	24.32%	24.44%	0.00%	0.00%	0.045
**Second episode**					
Mean days of positivity (Mean, SD)	10.1 (10.5)	11.2 (11.1)	5.7 (6)	16.1 (12.7)	0.238
Mild COVID-19	76.67%	36.59%	66.67%	92.86%	0.005
Moderate COVID-19	10.00%	34.15%	33.33%	7.14%	
Severe COVID-19	3.33%	14.63%	0.00%	0.00%	
Critical COVID-19	10.00%	14.63%	0.00%	0.00%	
Hospitalization	82.5%	83.3%	55.6%	64.3%	0.135
Pneumonia	87.5%	96.6%	50.0%	0.0%	0.001
Fever	25.81%	37.21%	33.33%	14.29%	0.417
Cough	33.33%	34.15%	25.00%	14.29%	0.505
Dyspnea	16.13%	44.19%	8.33%	14.29%	0.011
Arthromyalgia	12.90%	23.81%	33.33%	7.14%	0.257
Headache	3.23%	13.95%	8.33%	14.29%	0.423
General cold symptoms	12.90%	18.60%	8.33%	21.43%	0.778
Asthenia	3.23%	9.30%	8.33%	14.29%	0.487
Gastrointestinal symptoms	6.45%	13.95%	8.33%	0.00%	0.521

COPD: chronic obstructive pulmonary disease; COVID-19: Coronavirus disease 2019; SD: standard deviation; T2DM: type 2 diabetes mellitus.

## Data Availability

The original contributions generated for the study are included in the article/Supplementary Material, further inquiries can be directed to the corresponding author/s.

## Supplementary Materials

The following are available online at https://www.mdpi.com/1999-4915/13/3/512/s1, Table S1: PRISMA checklist, Table S2: Classification of COVID-19 severity, Table S3: Data quality of the analyzed cohort, Table S4: Basic characteristics of the included case reports, Table S5: Basic characteristics of the included case series, Table S6: Difference of intervals organized by disease severity, Table S7: Repeated SARS-CoV-2 positive intervals based on symptomatic cases, Table S8: Risk of bias assessment for case reports, Table S9: Risk of bias assessment for case series, Figure S1: PRISMA flowchart, Figure S2: Histogram of analyzed intervals, Figure S3: Correlation analysis.
