# Supplementary material for: Repeated SARS-CoV-2 Positivity: Analysis of 123 Cases
Source: Viruses. 2021 Mar 19;13(3):512. doi: 10.3390/v13030512 (PMC8003803; doi:10.3390/v13030512)
Supplement: Supplementary file 1 [file viruses-13-00512-s001.pdf]

Table S1. PRISMA checklist [1].

Section/topic	#	Checklist item	Reported on page #
**TITLE**			
Title	1	Identify the report as a systematic review, meta-analysis, or both.	1
**ABSTRACT**			
Structured summary	2	Provide a structured summary including, as applicable: background; objectives; data sources; study eligibility criteria, participants, and interventions; study appraisal and synthesis methods; results; limitations; conclusions and implications of key findings; systematic review registration number.	3
**INTRODUCTION**			
Rationale	3	Describe the rationale for the review in the context of what is already known.	4
Objectives	4	Provide an explicit statement of questions being addressed with reference to participants, interventions, comparisons, outcomes, and study design (PICOS).	4
**METHODS**			
Protocol and registration	5	Indicate if a review protocol exists, if and where it can be accessed (e.g., Web address), and, if available, provide registration information including registration number.	5
Eligibility criteria	6	Specify study characteristics (e.g., PICOS, length of follow-up) and report characteristics (e.g., years considered, language, publication status) used as criteria for eligibility, giving rationale.	5
Information sources	7	Describe all information sources (e.g., databases with dates of coverage, contact with study authors to identify additional studies) in the search and date last searched.	5
Search	8	Present full electronic search strategy for at least one database, including any limits used, such that it could be repeated.	5
Study selection	9	State the process for selecting studies (i.e., screening, eligibility, included in systematic review, and, if applicable, included in the meta-analysis).	5
Data collection process	10	Describe method of data extraction from reports (e.g., piloted forms, independently, in duplicate) and any processes for obtaining and confirming data from investigators.	5
Data items	11	List and define all variables for which data were sought (e.g., PICOS, funding sources) and any assumptions and simplifications made.	5
Risk of bias in individual studies	12	Describe methods used for assessing risk of bias of individual studies (including specification of whether this was done at the study or outcome level), and how this information is to be used in any data synthesis.	6

Section/topic	#	Checklist item	Reported on page #
Summary measures	13	State the principal summary measures (e.g., risk ratio, difference in means).	5-6
Synthesis of results	14	Describe the methods of handling data and combining results of studies, if done, including measures of consistency (e.g., I^2) for each meta-analysis.	5-6
Risk of bias across studies	15	Specify any assessment of risk of bias that may affect the cumulative evidence (e.g., publication bias, selective reporting within studies).	5-6
Additional analyses	16	Describe methods of additional analyses (e.g., sensitivity or subgroup analyses, meta-regression), if done, indicating which were pre-specified.	5
**RESULTS**			
Study selection	17	Give numbers of studies screened, assessed for eligibility, and included in the review, with reasons for exclusions at each stage, ideally with a flow diagram.	7
Study characteristics	18	For each study, present characteristics for which data were extracted (e.g., study size, PICOS, follow-up period) and provide the citations.	7
Risk of bias within studies	19	Present data on risk of bias of each study and, if available, any outcome level assessment (see item 12).	9
Results of individual studies	20	For all outcomes considered (benefits or harms), present, for each study: (a) simple summary data for each intervention group (b) effect estimates and confidence intervals, ideally with a forest plot.	7-8
Synthesis of results	21	Present results of each meta-analysis done, including confidence intervals and measures of consistency.	7-8
Risk of bias across studies	22	Present results of any assessment of risk of bias across studies (see Item 15).	-
Additional analysis	23	Give results of additional analyses, if done (e.g., sensitivity or subgroup analyses, meta-regression [see Item 16]).	7-9
**DISCUSSION**			
Summary of evidence	24	Summarize the main findings including the strength of evidence for each main outcome; consider their relevance to key groups (e.g., healthcare providers, users, and policy makers).	10
Limitations	25	Discuss limitations at study and outcome level (e.g., risk of bias), and at review-level (e.g., incomplete retrieval of identified research, reporting bias).	11
Conclusions	26	Provide a general interpretation of the results in the context of other evidence, and implications for future research.	12
**FUNDING**			

Section/topic	#	Checklist item	Reported on page #
Funding	27	Describe sources of funding for the systematic review and other support (e.g., supply of data); role of funders for the systematic review.	2

Table S2. Classification of COVID-19 severity [2].

Severity	Definition
Mild	The clinical symptoms are mild with no abnormal radiological findings.
Moderate	Fever, cough, and other symptoms are presented with pneumonia on chest computed tomography.
Severe	One of the following conditions is met: (1) Respiratory distress, respiratory rate ≥ 30 per min; (2) Oxygen saturation on room air at rest ≤ 93%; (3) Partial pressure of oxygen in arterial blood / fraction of inspired oxygen ≤ 300 mmHg.
Critical	One of the following conditions has to be met: (1) Respiratory failure occurs and mechanical ventilation is required; (2) Shock occurs; (3) Patients with other organ dysfunction needing intensive care unit monitoring.

Table S3. Data quality of the analyzed cohort

Parameter		Total	Data	%
Country		123	123	100.00%
Gender		123	122	99.19%
Age		123	120	97.56%
Comorbidities				
	Hypertension	123	82	66.67%
	Chronic heart disease	123	84	68.29%
	Arrhythmia	123	84	68.29%
	Type 2 diabetes mellitus	123	82	66.67%
	Chronic obstructive lung disease	123	82	66.67%
	Chronic kidney disease	123	82	66.67%
	Chronic liver disease	123	82	66.67%
	Immunosuppression	123	82	66.67%
	Cancer	123	84	68.29%
	Other	123	78	63.41%
Chronic treatment		123	22	17.89%
Smoking status		123	16	13.01%
Current alcohol consumption		123	14	11.38%
Body mass index		123	7	5.69%
First episode characteristics				
	Symptoms start date	123	97	78.86%
	Symptoms end date	123	52	42.28%
	First positive PCR date	123	123	100.00%
	First negative PCR date	123	97	78.86%
	Severity	123	109	88.62%
	Fever	123	108	87.80%
	Cough	123	108	87.80%
	Dyspnea	123	105	85.37%
	O2 saturation	123	23	18.70%
	Arthromyalgia	123	108	87.80%
	Headache	123	108	87.80%

	General cold symptoms	123	108	87.80%
	Asthenia	123	109	88.62%
	Gastrointestinal symptoms	123	109	88.62%
	Other	123	110	89.43%
	Hospitalization	123	119	96.75%
	Department	123	45	36.59%
	Days of hospitalization	123	97	78.86%
	COVID-19 Treatment	123	99	80.49%
	Chest X-ray	123	19	15.45%
	Chest computed tomography	123	55	44.72%
	Pneumonia	123	62	50.41%
	Abdominal imaging	123	10	8.13%
	Any laboratory measurement	123	66	53.66%
	White blood cell count	123	38	30.89%
	Lymphocyte count	123	47	38.21%
	Lactate dehydrogenase	123	27	21.95%
	C-reactive protein	123	45	36.59%
	Procalcitonin	123	30	24.39%
	Thrombocyte	123	28	22.76%
	IgM status	123	21	17.07%
	IgG status	123	36	29.27%
Second episode characteristics				
	Symptoms start date	123	92	74.80%
	Symptoms end date	123	67	54.47%
	First positive PCR date	123	121	98.37%
	First negative PCR date	123	59	47.97%
	Severity	123	97	78.86%
	Fever	123	100	81.30%
	Cough	123	97	78.86%
	Dyspnea	123	100	81.30%
	O2 saturation	123	16	13.01%
	Arthromyalgia	123	99	80.49%
	Headache	123	100	81.30%
	General cold symptoms	123	100	81.30%
	Asthenia	123	100	81.30%
	Gastrointestinal symptoms	123	100	81.30%
	Other	123	101	82.11%
	Hospitalization	123	111	90.24%
	Department	123	47	38.21%
	Days of hospitalization	123	66	53.66%
	COVID-19 Treatment	123	67	54.47%
	Chest X-ray	123	14	11.38%
	Chest computed tomography	123	55	44.72%
	Pneumonia	123	61	49.59%
	Abdominal imaging	123	0	0.00%
	Any laboratory measurement	123	49	39.84%

White blood cell count	123	23	18.70%
Lymphocyte count	123	39	31.71%
Lactate dehydrogenase	123	11	8.94%
C-reactive protein	123	32	26.02%
Procalcitonin	123	7	5.69%
Thrombocyte	123	8	6.50%
IgM status	123	25	20.33%
IgG status	123	52	42.28%

Table S4. Basic characteristics of included case reports

Author	Country	Sex	Age	Repeated positivity length
Alfano G et al., 2020 [3]	Italy	Male	72	35
AlFehaidi A et al., 2020 [4]	Qatar	Female	46	80
Alonso FOM et al., 2021 [5]	Brazil	Male	26	46
Bentivegna E et al., 2021 [6]	Italy	Female	62	46
Bongiovani M et al., 2020 [7]	Italy	F/F/F	81/ 85/ 48	32/ 28/ 117
Bonifacio LP et al., 2020[8]	Brazil	Female	24	140
Chen D et al., 2020 [9]	China	Female	46	18
Colson P et al., 2020 [10]	France	Male	70	119
Coppola A et al., 2020 [11]	Italy	Male	68	36
de Brito CAA et al., 2020 [12]	Brazil	Male/ Female	40/ 44	44/ 25
Dou C et al., 2020 [13]	China	Male	34	34
Duggan NM et al., 2021 [14]	USA	Male	82	48
Fehdi AM et al., 2020 [15]	Morocco	Male	69	18
Fu W et al., 2020 [16]	China	Female/ Male /Female	36/ 74/ 34	35/ 36/ 28
Geling T et al., 2020 [17]	China	Male	24	20
Goldman DJ et al., 2020 [18]	USA	ND	65	140
Gupta V et al., 2020 [19]	India	M/F	25/ 28	108/ 111
He F et al., 2020 [20]	China	Female	39	51
Jiang M et al., 2020 [21]	China	Female/ Female	35/ 56	27/ 28
Lafaie L et al., 2020 [22]	France	Female/ Male /Female	84/ 90/ 84	40/ 36/ 20
Larson D et al., 2020 [23]	USA	Male	42	65
Leipe J et al., 2020 [24]	Germany	Male	63	14
Li J et al., 2020 [25]	China	Male	50	34
Liu F et al., 2020 [26]	China	Female	57	24
Liu F et al., 2020 [27]	China	Male	35	30
Loconsole D et al., 2020 [28]	Italy	Male	48	43
Luo A et al., 2020 [29]	China	Female	58	36
Ma BM et al., 2020 [30]	Japan	Female	31	20
Marchev S et al., 2020 [31]	Bulgaria	Male	25	92
Mardani M et al., 2020 [32]	Iran	Female	64	35
Mendoza JM et al., 2020 [33]	USA	Male	51	60

Mulder M et al., 2020 [34]	Netherlands	Female	89	59
Nachmias V et al., 2020 [35]	Israel	Female	20	111
Nazir N et al., 2020 [36]	India	Male	26	110
Ogawa Y et al., 2020 [37]	Japan	Female	81	44
Qiao X et al., 2020 [38]	China	Female	30	29
Ravioli S et al., 2020 [39]	Switzerland	Female/ Female	81/ 77	36/ 30
Thomas SV et al., 2020 [40]	United Arab Emirates	Male	39	43
Tillett RL et al., 2021 [41]	USA	Male	25	48
To KKW et al., 2020 [42]	China	Male	33	142
van Elslande J et al., 2020 [43]	Belgium	Female	51	93
Wang P et al., 2021 [44]	China	Male	33	84
Wei L et al., 2020 [45]	China	Male	61	61
Yoo SY et al., 2020 [46]	China	Male	8	35
Yuan B et al., 2020 [47]	China	Male	8	30
Zanardini C et al., 2020 [48]	Italy	Female/ Female	33/ 27	119/ 91
Zhou X et al., 2020 [49]	China	Male	40	25

Table S5. Basic characteristics of the included case series

**Author.**	**Country**	**Nr of patients (female %)**	**Mean age (SD)**	**Mean repeated positivity length (SD)**
Chen Y et al., 2020 [50]	China	4 (50)	32 (15.6)	21 (7.1)
Fernandes VTC et al., 2020 [51]	Brazil	6 (16.7)	46.2 (16)	56.8 (9.7)
Gidari A et al., 2020 [52]	Italy	9 (33.3)	54.7 (17.7)	60.9 (23.3)
Goussef M et al., 2020 [53]	France	11 (45.5)	58.3 (24.5)	35.2 (8.8)
Li Y et al., 2020 [54]	China	4 (50)	62.3 (17.5)	32.3 (20.8)
Peng J et al., 2020 [55]	China	6 (50)	41.3 (22.2)	30.2 (9.3)
Tao W et al., 2020 [56]	China	12 (41.7)	48.2 (14)	39.5 (26.1)
Tomassini S et al., 2020 [57]	UK	6 (50)	75.8 (16.7)	66 (19.1)
Zhang B et al., 2020 [58]	China	7 (14.3)	22.4 (13.3)	23.4 (4.8)

Table S6. Difference of intervals organized by disease severity

**Intervals (n/total)**	**Mild Mean (SD), Median (Q1-Q3)**	**Moderate Mean (SD), Median (Q1-Q3)**	**Severe Mean (SD), Median (Q1-Q3)**	**Critical Mean (SD), Median (Q1-Q3)**	**p-value**
		**1st episode severity**			
**RSP** (123/123)	51.35 (32.87)<br>37 (27-65)	54.03 (34.70)<br>42.5 (28.5-70.5)	41.06 (20.57)<br>34.5 (27-52.5)	34.43 (11.46)<br>36 (20-44)	0.537
**NTP** (94/123)	32.85 (32.23)<br>17 (11.5-46)	33.06 (31.47)<br>20 (11-46)	17.85 (11.40)<br>14 (10-21)	17.17 (9.09)<br>19 (9-24)	0.616
**STS** (50/123)	48.50 (27.16)<br>43 (25-73)	63.35 (29.27)<br>58 (46-70)	44.00 (23.11)<br>32.5 (28.5-55)	36.83 (14.73)<br>40.5 (20-48)	0.091
**NSTS** (33/123)	28.20 (24.99)<br>37 (2-43)	35.89 (23.88)<br>32 (18-52)	22.80 (23.61)<br>10 (8-36)	18.67 (9.07)<br>20 (9-27)	0.736
**NSTP** (46/123)	40.29 (22.57)	27.79 (18.02)	21.50 (21.35)	19.00 (9.54)	0.291

	43 (23-60)	29 (11-40)	12.5 (8-36)	20 (9-28)	
		2nd episode severity			
**RSP** (123/123)	55.12 (37.82)<br>36 (26-84)	48.55 (18.73)<br>43.5 (36-59)	47.86 (13.13)<br>54 (35-59)	33.89 (11.40)<br>36 (23-44)	0.377
**NTP** (94/123)	34.17 (33.98)<br>17 (10.5-52.5)	31.29 (17.70)<br>25.5 (20-45)	24.00 (11.69)<br>24 (15.5-32.5)	15.80 (9.44)<br>18 (9-20)	0.486
**STS** (50/123)	61.17 (37.81)<br>49 (29.5-91)	51.75 (22.46)<br>48 (41-62.5)	49.83 (15.14)<br>56.5 (35-60)	35.56 (12.89)<br>40 (27-45)	0.188
**NSTS** (33/123)	27.50 (26.27)<br>25.5 (2-52)	31.36 (23.24)<br>32 (15-40)	31.67 (22.01)<br>31 (10-54)	12.00 (9.32)<br>7.5 (6-20)	0.412
**NSTP** (46/123)	26.58 (21.48)<br>15 (8-43)	32.73 (16.68)<br>32 (23-42)	34.33 (22.37)<br>39 (10-54)	12.17 (9.97)<br>9.5 (4-20)	0.120

NSTP: no-symptom to positive PCR interval; NSTS: no-symptom to symptom interval; NTP: negative to positive PCR interval; PCR: polymerase chain reaction; PTN: positive to negative PCR interval; Q1-Q3: 25 and 75% percentiles; RSP: repeatedly SARS-CoV-2 positivity; SD: standard deviation; STS: symptom to symptom interval

Table S7. Repeated SARS-CoV-2 positive intervals based on symptomatic cases

	**Mean (SD) interval**	**Median (Q1-Q3) interval**
All included patients	47.9 (30.1) days	36 (28-49) days
Asymptomatic 1st episode	75.3 (39.7) days	84 (43-110) days
Asymptomatic 2nd episode	58.7 (35.7) days	43 (28-84) days
Both episode asymptomatic	78.4 (43.6) days	108 (31-110) days

Q1-Q3: 25 and 75% percentiles; SD: standard deviation

Table S8. Risk of bias assessment using the CARE tool [59] for case reports

Author	1	2	3	4	5	6	7	8
Alfano G et al., 2020 [3]								
AlFehaidi A et al., 2020 [4]								
Alonso FOM et al., 2021 [5]								
Bentivegna E et al., 2021 [6]								
Bongiovani M et al., 2020 [7]								
Bonifacio LP et al., 2020[8]								
Chen D et al., 2020 [9]								
Colson P et al., 2020 [10]								
Coppola A et al., 2020 [11]								
de Brito CAA et al., 2020 [12]								
Dou C et al., 2020 [13]								
Duggan NM et al., 2021 [14]								
Fehdi AM et al., 2020 [15]								
Fu W et al., 2020 [16]								
Geling T et al., 2020 [17]								
Goldman DJ et al., 2020 [18]								
Gupta V et al., 2020 [19]								
He F et al., 2020 [20]								
Jiang M et al., 2020 [21]								
Lafaie L et al., 2020 [22]								
Larson D et al., 2020 [23]								
Leipe J et al., 2020 [24]								
Li J et al., 2020 [25]								
Liu F et al., 2020 [26]								
Liu F et al., 2020 [27]								
Loconsole D et al., 2020 [28]								
Luo A et al., 2020 [29]								
Ma BM et al., 2020 [30]								
Marchev S et al., 2020 [31]								
Mardani M et al., 2020 [32]								
Mendoza JM et al., 2020 [33]								
Mulder M et al., 2020 [34]								
Nachmias V et al., 2020 [35]								
Nazir N et al., 2020 [36]								
Ogawa Y et al., 2020 [37]								
Qiao X et al., 2020 [38]								
Ravioli S et al., 2020 [39]								
Thomas SV et al., 2020 [40]								
Tillett RL et al., 2021 [41]								
To KKW et al., 2020 [42]								
van Elslande J et al., 2020 [43]								
Wang P et al., 2021 [44]								
Wei L et al., 2020 [45]								

Yoo SY et al., 2020 [46]								
Yuan B et al., 2020 [47]								
Zanardini C et al., 2020 [48]								
Zhou X et al., 2020 [49]								

When assessing risk of bias in case reports, the following questions were answered:

1. Were patient's demographic characteristics clearly described? 2. Was the patient's history clearly described and presented as a timeline? 3. Was the current clinical condition of the patient on presentation clearly described? 4. Were diagnostic tests or methods and the results clearly described? 5. Was the intervention(s) or treatment procedure(s) clearly described? 6. Was the post-intervention clinical condition clearly described? 7. Were adverse events (harms) or unanticipated events identified and described? 8. Does the case report provide takeaway lessons?

Based on the CARE guideline [59], each domain was assessed as low (green), unclear (yellow) or high (red) risk of bias based on the available descriptions in each included study.

Table S9. Risk of bias assessment using the CARE tool [59] for case series

Author	1	2	3	4	5	6	7	8	9	10
Chen Y et al., 2020 [50]										N/A
Fernandes VTC et al., 2020 [51]										N/A
Gidari A et al., 2020 [52]										N/A
Goussef M et al., 2020 [53]										N/A
Li Y et al., 2020 [54]										N/A
Peng J et al., 2020 [55]										N/A
Tao W et al., 2020 [56]										N/A
Tomassini S et al., 2020 [57]										N/A
Zhang B et al., 2020 [58]										N/A

When assessing risk of bias in case series, the following questions were answered:

1. Were there clear criteria for inclusion in the case series? 2. Was the condition measured in a standard, reliable way for all participants included in the case series? 3. Were valid methods used for identification of the condition for all participants included in the case series? 4. Did the case series have consecutive inclusion of participants? 5. Did the case series have complete inclusion of participants? 6. Was there clear reporting of the demographics of the participants in the study? 7. Was there clear reporting of clinical information of the participants? 8. Were the outcomes or follow up results of cases clearly reported? 9. Was there clear reporting of the presenting site(s)/clinic(s) demographic information? 10. Was statistical analysis appropriate?

Based on the CARE guideline [59], each domain was assessed as low (green), unclear (yellow) or high (red) risk of bias based on the available descriptions in each included study. The last question was not assessed since statistical analysis was not relevant for the included case series.

Table S10. Summary of reported SARS-CoV-2 strains

	Case number	1st episode strain	2nd episode strain
Goldman DJ et al [18]	Reinf_26	clade 19B	clade 20A<br>A23403G mutation, which confers the D614G amino acid change in spike protein
Gupta V et al [19]	Reinf_27<br>Reinf_28	both the genomes revealed 9 and 10 unique variant differences between the virus isolates from the 2 episodes of infection	
Colson P et al [10]	Reinf_33	Nextrain clade 20A	Marseille 4 lineage<br>11 mutations that are hallmarks of the Marseille 4 lineage (C4543U, G5629U, G9526U, C11497U, G13993U, G15766U, A16889G, G17019U, G22992A, G28975C, G29399A) were absent from the genome obtained from the first sample. In contrast, 2 mutations (C2416U, G8371U) that are hallmarks of the genotype identified in the first sample were absent in the second genome.
Tillett RL et al [41]	Reinf_47	Both strains were members of clade 20C. Specimen A had five further SNVs compared with the reference genome. Specimen B showed six additional SNVs and a mutation at position 14 407, adjacent to the SNV 14408C→T and recorded as a dinucleotide multinucleotide variant (MNV) at positions 14 407 and 14 408 of the genome. Six SNVs were shared between specimen A and specimen B (table 2). Specimen A had four additional SNVs not seen in specimen B, whereas specimen B had seven SNVs that were absent in specimen A.	
To KKW et al [42]	Reinf_48	lineage B.2	lineage B.1.79
van Elslande J et al [43]	Reinf_49	lineage B.1.1	lineage A
Larson D et al [23]	Reinf_95	lineage B.1.26 and the genome encoded the D614G variation in the spike protein	several potential variations
Mulder M et al [34]	Reinf_109	The two strains differed at ten nucleotide positions in the ORF1a (4), ORF (2), Spike (2), ORF3a (1) and M (1) genes	

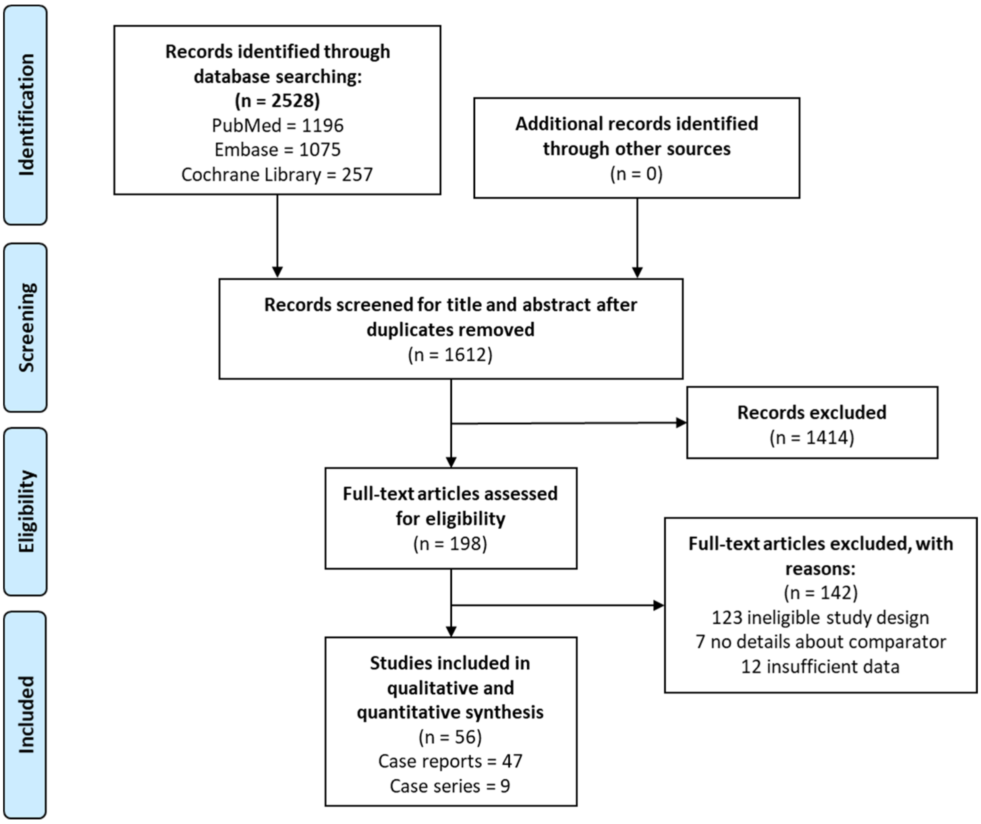

Figure S1. Preferred Reporting Items for Systematic Reviews and Meta-Analyses checklist

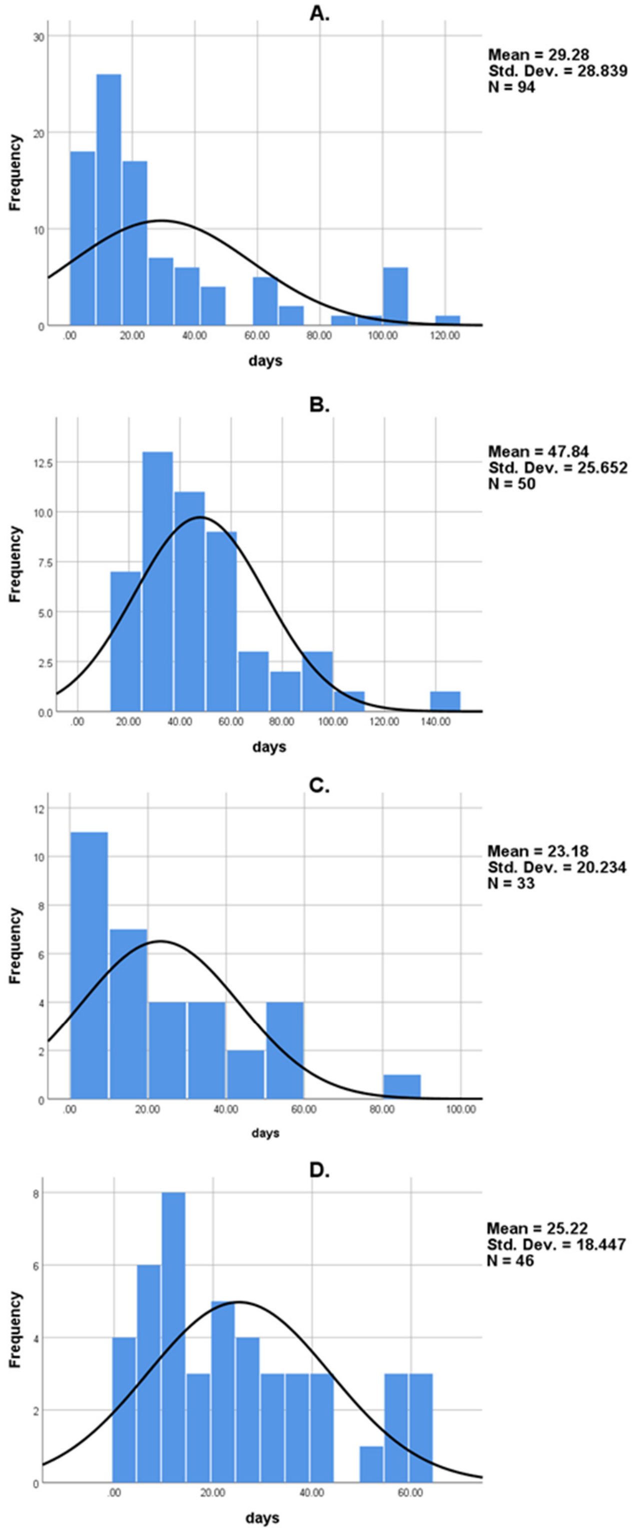

Figure S2. Histogram of analyzed intervals. (A) negative to positive polymerase chain reaction interval, (B) symptom to symptom interval, (C) no symptom to symptom, (D) no symptom to positive polymerase chain reaction interval

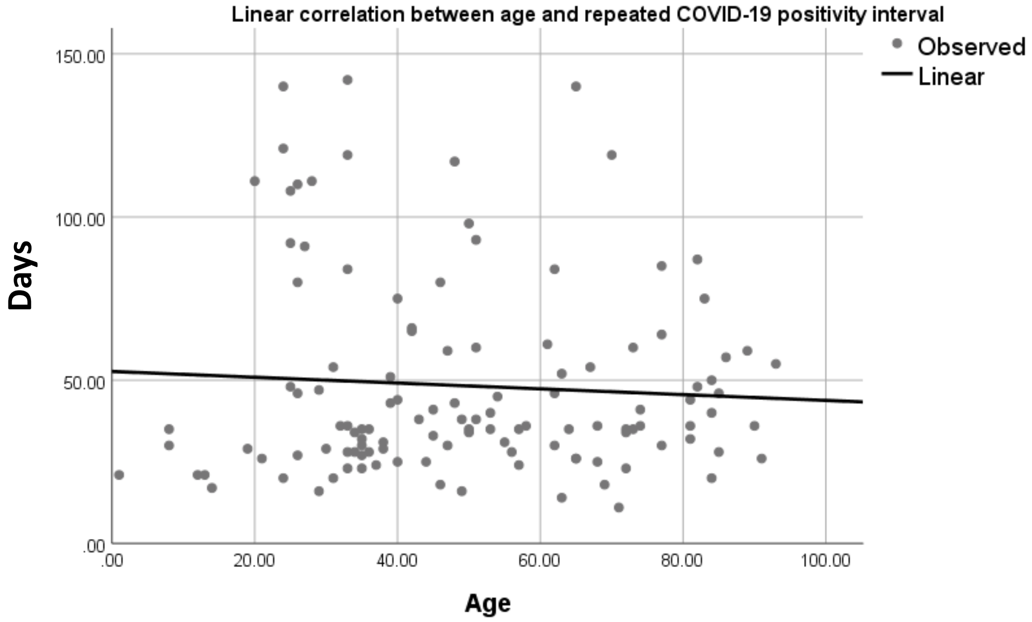

Figure S3. Correlation analysis between age (years) and repeated positivity length (days)

REFERENCES

1. Moher, D.; Liberati, A.; Tetzlaff, J.; Altman, D.G. Preferred reporting items for systematic reviews and meta-analyses: the PRISMA statement. *PLoS medicine* **2009**, *6*, e1000097, doi:10.1371/journal.pmed.1000097.
2. Zu, Z.Y.; Jiang, M.D.; Xu, P.P.; Chen, W.; Ni, Q.Q.; Lu, G.M.; Zhang, L.J. Coronavirus Disease 2019 (COVID-19): A Perspective from China. *Radiology* **2020**, *296*, E15-e25, doi:10.1148/radiol.2020200490.
3. Alfano, G.; Perrone, R.; Fontana, F.; Mori, G.; Lucchi, L.; Guaraldi, G.; Magistroni, R.; Cappelli, G.; for the Modena Covid-19 Working, G. Long-term effects of COVID-19 in a patient on maintenance dialysis. *Hemodial Int* **2020**, *24*, E50-E54, doi:10.1111/hdi.12859.
4. AlFehaidi, A.; Ahmad, S.A.; Hamed, E. SARS-CoV-2 re-infection: a case report from Qatar. *J Infect* **2020**, 10.1016/j.jinf.2020.10.019, doi:10.1016/j.jinf.2020.10.019.
5. Alonso, F.O.M.; Sabino, B.D.; Guimarães, M.A.A.M.; Varella, R.B. Recurrence of SARS-CoV-2 infection with a more severe case after mild COVID-19, reversion of RT-qPCR for positive and late antibody response: Case report. *J Med Virol* **2021**, *93*, 655-656, doi:10.1002/jmv.26432.
6. Bentivegna, E.; Sentimentale, A.; Luciani, M.; Speranza, M.L.; Guerritore, L.; Martelletti, P. New IgM seroconversion and positive RT-PCR test after exposure to the virus in recovered COVID-19 patient. *J Med Virol* **2021**, *93*, 97-98, doi:10.1002/jmv.26160.
7. Bongiovanni, M.; Basile, F. Re-infection by COVID-19: a real threat for the future management of pandemia? *Infect Dis* **2020**, *52*, 581-582, doi:10.1080/23744235.2020.1769177.
8. Bonifácio, L.P.; Pereira, A.P.S.; Araújo, D.C.d.A.e.; Balbão, V.d.M.P.; Fonseca, B.A.L.d.; Passos, A.D.C.; Bellissimo-Rodrigues, F. Are SARS-CoV-2 reinfection and Covid-19 recurrence possible? a case report from Brazil. *Rev Soc Bras Med Trop* **2020**, *53*, e20200619, doi:10.1590/0037-8682-0619-2020.
9. Chen, D.; Xu, W.; Lei, Z.; Huang, Z.; Liu, J.; Gao, Z.; Peng, L. Recurrence of positive SARS-CoV-2 RNA in COVID-19: A case report. *Int J Infect Dis* **2020**, *93*, 297-299, doi:10.1016/j.ijid.2020.03.003.
10. Colson, P.; Finaud, M.; Levy, N.; Lagier, J.-C.; Raoult, D. Evidence of SARS-CoV-2 re-infection with a different genotype. *J Infect* **2020**, 10.1016/j.jinf.2020.11.011, doi:10.1016/j.jinf.2020.11.011.
11. Coppola, A.; Annunziata, A.; Carannante, N.; Di Spirito, V.; Fiorentino, G. Late Reactivation of SARS-CoV-2: A Case Report. *Front Med* **2020**, *7*, 531, doi:10.3389/fmed.2020.00531.
12. de Brito, C.A.A.; Lima, P.M.A.; de Brito, M.C.M.; de Oliveira, D.B. Second Episode of COVID-19 in Health Professionals: Report of Two Cases. *Int Med Case Rep J* **2020**, *13*, 471-475, doi:10.2147/imcrj.S277882.
13. Dou, C.; Xie, X.; Peng, Z.; Tang, H.; Jiang, Z.; Zhong, Z.; Tang, J. A case presentation for positive SARS-CoV-2 RNA recurrence in a patient with a history of type 2 diabetes that had recovered from severe COVID-19. *Diabetes Res Clin Pract* **2020**, *166*, 108300, doi:10.1016/j.diabres.2020.108300.
14. Duggan, N.M.; Ludy, S.M.; Shannon, B.C.; Reisner, A.T.; Wilcox, S.R. Is novel coronavirus 2019 reinfection possible? Interpreting dynamic SARS-CoV-2 test results. *Am J Emerg Med* **2021**, *39*, 256.e251-256.e253, doi:10.1016/j.ajem.2020.06.079.
15. Fehdi, M.A.; Erragh, A.; Zerhouni, A.; Aissaoui, O.; Nsiri, A.; Alharrar, R. Case report: a COVID-19 reactivation case. *Pan Afr Med J* **2020**, *35*, doi:10.11604/pamj.2020.35.2.23326.
16. Fu, W.; Chen, Q.; Wang, T. Letter to the Editor: Three cases of redetectable positive SARS-CoV-2 RNA in recovered COVID-19 patients with antibodies. *J Med Virol* **2020**, *92*, 2298-2301, doi:10.1002/jmv.25968.
17. Geling, T.; Huaizheng, G.; Ying, C.; Hua, H. Recurrent positive nucleic acid detection in a recovered COVID-19 patient: A case report and literature review. *Respir Med Case Rep* **2020**, *31*, 101152, doi:10.1016/j.rmcr.2020.101152.
18. Goldman, J.D.; Wang, K.; Roltgen, K.; Nielsen, S.C.A.; Roach, J.C.; Naccache, S.N.; Yang, F.; Wirz, O.F.; Yost, K.E.; Lee, J.-Y., et al. Reinfection with SARS-CoV-2 and Failure of Humoral Immunity: a case report. *medRxiv* **2020**, 10.1101/2020.09.22.20192443, 2020.2009.2022.20192443, doi:10.1101/2020.09.22.20192443.

19. Gupta, V.; Bhoyar, R.C.; Jain, A.; Srivastava, S.; Upadhayay, R.; Imran, M.; Jolly, B.; Divakar, M.K.; Sharma, D.; Sehgal, P., et al. Asymptomatic Reinfection in 2 Healthcare Workers From India With Genetically Distinct Severe Acute Respiratory Syndrome Coronavirus 2. *Clin Infect Dis* **2020**, 10.1093/cid/ciaa1451, doi:10.1093/cid/ciaa1451.
20. He, F.; Luo, Q.; Lei, M.; Fan, L.; Shao, X.; Hu, K.; Qin, S.; Yu, N.; Cao, J.; Yang, L. Successful recovery of recurrence of positive SARS-CoV-2 RNA in COVID-19 patient with systemic lupus erythematosus: a case report and review. *Clin Rheumatol* **2020**, *39*, 2803-2810, doi:10.1007/s10067-020-05230-0.
21. Jiang, M.; Li, Y.; Han, M.; Wang, Z.; Zhang, Y.; Du, X. Recurrent PCR positivity after hospital discharge of people with coronavirus disease 2019 (COVID-19). *J Infect* **2020**, *81*, 147-178, doi:10.1016/j.jinf.2020.03.024.
22. Lafaie, L.; Célarier, T.; Goethals, L.; Pozzetto, B.; Grange, S.; Ojardias, E.; Annweiler, C.; Botelho-Nevers, E. Recurrence or Relapse of COVID-19 in Older Patients: A Description of Three Cases. *J Am Geriatr Soc* **2020**, *68*, 2179-2183, doi:10.1111/jgs.16728.
23. Larson, D.; Brodniak, S.L.; Voegtly, L.J.; Cer, R.Z.; Glang, L.A.; Malagon, F.J.; Long, K.A.; Potocki, R.; Smith, D.R.; Lanteri, C., et al. A Case of Early Reinfection With Severe Acute Respiratory Syndrome Coronavirus 2 (SARS-CoV-2). *Clin Infect Dis* **2020**, 10.1093/cid/ciaa1436, doi:10.1093/cid/ciaa1436.
24. Leipe, J.; Wilke, E.L.; Ebert, M.P.; Teufel, A.; Reindl, W. Long, relapsing, and atypical symptomatic course of COVID-19 in a B-cell-depleted patient after rituximab. *Semin Arthritis Rheum* **2020**, *50*, 1087-1088, doi:10.1016/j.semarthrit.2020.06.013.
25. Li, J.; Long, X.; Fang, X.; Zhang, Q.; Hu, S.; Lin, Z.; Xiong, N. SARS-CoV-2 positivity in a discharged COVID-19 patient: a case report. *Clin Microbiol Infect* **2020**, *26*, 1115-1117, doi:10.1016/j.cmi.2020.04.032.
26. Liu, F.; Cai, Z.-B.; Huang, J.-S.; Yu, W.-Y.; Niu, H.-Y.; Zhang, Y.; Sui, D.-M.; Wang, F.; Xue, L.-Z.; Xu, A.-F. Positive SARS-CoV-2 RNA recurs repeatedly in a case recovered from COVID-19: dynamic results from 108 days of follow-up. *Pathog Dis* **2020**, *78*, ftaa031, doi:10.1093/femspd/ftaa031.
27. Liu, F.; Cai, Z.-b.; Huang, J.-s.; Niu, H.-y.; Yu, W.-y.; Zhang, Y.; Yan, T.-b.; Chen, C.; Liu, Y.; Xu, A.-f. Repeated COVID-19 relapse during post-discharge surveillance with viral shedding lasting for 67 days in a recovered patient infected with SARS-CoV-2. *J Microbiol Immunol Infect* **2020**, 10.1016/j.jmii.2020.07.017, doi:10.1016/j.jmii.2020.07.017.
28. Loconsole, D.; Passerini, F.; Palmieri, V.O.; Centrone, F.; Sallustio, A.; Pugliese, S.; Grimaldi, L.D.; Portincasa, P.; Chironna, M. Recurrence of COVID-19 after recovery: a case report from Italy. *Infection* **2020**, *48*, 965-967, doi:10.1007/s15010-020-01444-1.
29. Luo, A. Positive SARS-Cov-2 test in a woman with COVID-19 at 22 days after hospital discharge: A case report. *J Tradit Chin Med* **2020**, *7*, 413-417, doi:10.1016/j.jtcms.2020.04.001.
30. Ma, B.M.; Hung, I.F.N.; Chan, G.C.W.; Tam, A.R.; Chan, S.S.K.; Wong, B.C.K.; Fukuda, K.; Ohno, T.; Yuen, K.Y.; Chan, T.M. Case of "relapsing" COVID-19 in a kidney transplant recipient. *Nephrology* **2020**, *25*, 933-936, doi:10.1111/nep.13786.
31. Marchev, S.; Hadzhimineva, N.; Simidchiev, A. Clinical Case: SARS-CoV-2 Reactivation After a Heavy Workout. *Acta Medica Bulg* **2020**, *47*, 38-40, doi:10.2478/amb-2020-0033.
32. Mardani, M.; Nadji, S.A.; Sarhangipor, K.A.; Sharifi-Razavi, A.; Baziboroun, M. COVID-19 infection recurrence presenting with meningoencephalitis. *New Microbes New Infect* **2020**, *37*, 100732, doi:10.1016/j.nmni.2020.100732.
33. Munoz Mendoza, J.; Alcaide, M.L. COVID-19 in a patient with end-stage renal disease on chronic in-center hemodialysis after evidence of SARS-CoV-2 IgG antibodies. Reinfection or inaccuracy of antibody testing. *IDCases* **2020**, *22*, e00943, doi:10.1016/j.idcr.2020.e00943.
34. Mulder, M.; van der Vegt, D.S.J.M.; Oude Munnink, B.B.; GeurtsvanKessel, C.H.; van de Bovenkamp, J.; Sikkema, R.S.; Jacobs, E.M.G.; Koopmans, M.P.G.; Wegdam-Blans, M.C.A. Reinfection of Severe Acute Respiratory Syndrome Coronavirus 2 in an Immunocompromised Patient: A Case Report. *Clin Infect Dis* **2020**, 10.1093/cid/ciaa1538, doi:10.1093/cid/ciaa1538.
35. Nachmias, V.; Fusman, R.; Mann, S.; Koren, G. The first case of documented Covid-19 reinfection in Israel. *IDCases* **2020**, *22*, e00970, doi:10.1016/j.idcr.2020.e00970.

36. Nazir, N.; Ahirwar, A.; Jain, S. Reinfection in a healthcare worker with COVID-19 in a hospital in North India. *Anaesth Pain Intensive Care* **2020**, *24*, 572-572, doi:10.35975/apic.v24i5.1369.
37. Ogawa, Y.; Nishida, K.; Gohma, I.; Kasahara, K.; Yano, H. Assessing the effects of exposure to a SARS-CoV-2 re-positive patient in healthcare personnel. *BMC Res Notes* **2020**, *13*, 511, doi:10.1186/s13104-020-05365-y.
38. Qiao, X.-M.; Xu, X.-F.; Zi, H.; Liu, G.-X.; Li, B.-H.; Du, X.; Tian, Z.-H.; Liu, X.-Y.; Luo, L.-S.; Wang, X. Re-positive Cases of Nucleic Acid Tests in Discharged Patients With COVID-19: A Follow-Up Study. *Front Med* **2020**, *7*, 349, doi:10.3389/fmed.2020.00349.
39. Ravioli, S.; Ochsner, H.; Lindner, G. Reactivation of COVID-19 pneumonia: A report of two cases. *J Infect* **2020**, *81*, e72-e73, doi:10.1016/j.jinf.2020.05.008.
40. Thomas, S.V.; Nizam, A.; Iqbal, T. Can SARS-CoV-2 Viral RNA Continually Persist in an Asymptomatic COVID-19 Patient? *Dubai Med J* **2020**, *3*, 129-132, doi:10.1159/000510917.
41. Tillett, R.L.; Sevinsky, J.R.; Hartley, P.D.; Kerwin, H.; Crawford, N.; Gorzalski, A.; Laverdure, C.; Verma, S.C.; Rossetto, C.C.; Jackson, D., et al. Genomic evidence for reinfection with SARS-CoV-2: a case study. *Lancet Infect Dis* **2021**, *21*, 52-58, doi:10.1016/S1473-3099(20)30764-7.
42. To, K.K.-W.; Hung, I.F.-N.; Ip, J.D.; Chu, A.W.-H.; Chan, W.-M.; Tam, A.R.; Fong, C.H.-Y.; Yuan, S.; Tsoi, H.-W.; Ng, A.C.-K., et al. Coronavirus Disease 2019 (COVID-19) Re-infection by a Phylogenetically Distinct Severe Acute Respiratory Syndrome Coronavirus 2 Strain Confirmed by Whole Genome Sequencing. *Clin Infect Dis* **2020**, 10.1093/cid/ciaa1275, doi:10.1093/cid/ciaa1275.
43. Van Elslande, J.; Vermeersch, P.; Vandervoort, K.; Wawina-Bokalanga, T.; Vanmechelen, B.; Wollants, E.; Laenen, L.; André, E.; Van Ranst, M.; Lagrou, K., et al. Symptomatic SARS-CoV-2 reinfection by a phylogenetically distinct strain. *Clin Infect Dis* **2020**, 10.1093/cid/ciaa1330, doi:10.1093/cid/ciaa1330.
44. Wang, P. Recurrent presence of SARS-CoV-2 RNA in a 33-year-old man. *J Med Virol* **2021**, *93*, 592-594, doi:10.1002/jmv.26334.
45. Wei, L.; Liu, B.; Zhao, Y.; Chen, Z. Prolonged shedding of SARS-CoV-2 in an elderly liver transplant patient infected by COVID-19: a case report. *Ann Palliat Med* **2020**, 10.21037/apm-20-996, doi:10.21037/apm-20-996.
46. Yoo, S.Y.; Lee, Y.; Lee, G.H.; Kim, D.H. Reactivation of SARS-CoV-2 after recovery. *Pediatr Int* **2020**, *62*, 879-881, doi:10.1111/ped.14312.
47. Yuan, B.; Liu, H.-Q.; Yang, Z.-R.; Chen, Y.-X.; Liu, Z.-Y.; Zhang, K.; Wang, C.; Li, W.-X.; An, Y.-W.; Wang, J.-C., et al. Recurrence of positive SARS-CoV-2 viral RNA in recovered COVID-19 patients during medical isolation observation. *Sci Rep* **2020**, *10*, 11887, doi:10.1038/s41598-020-68782-w.
48. Zanardini, C.; Saccani, B.; Franceschetti, L.; Zatti, S.; Sartori, E.; Prefumo, F. Retest positive for SARS-CoV-2 RNA in pregnant women recovered from COVID-19. *Ultrasound Obstet Gynecol* **2020**, *56*, 948-949, doi:10.1002/uog.23144.
49. Zhou, X.; Zhou, J.; Zhao, J. Recurrent pneumonia in a patient with new coronavirus infection after discharge from hospital for insufficient antibody production: a case report. *BMC Infect Dis* **2020**, *20*, 500, doi:10.1186/s12879-020-05231-z.
50. Chen, Y.; Bai, W.; Liu, B.; Huang, J.; Laurent, I.; Chen, F.; Deng, W.; Xiao, X. Re-evaluation of retested nucleic acid-positive cases in recovered COVID-19 patients: Report from a designated transfer hospital in Chongqing, China. *J Infect Public Health* **2020**, *13*, 932-934, doi:10.1016/j.jiph.2020.06.008.
51. Fernandes Valente Takeda, C.; Moura de Almeida, M.; Gonçalves de Aguiar Gomes, R.; Cisne Souza, T.; Alves de Lima Mota, M.; Pamplona de Góes Cavalcanti, L.; Baima Colares, J.K. Case Report: Recurrent Clinical Symptoms of COVID-19 in Healthcare Professionals: A Series of Cases from Brazil. *Am J Trop Med Hyg* **2020**, *103*, 1993-1996, doi:10.4269/ajtmh.20-0893.
52. Gidari, A.; Nofri, M.; Saccarelli, L.; Bastianelli, S.; Sabbatini, S.; Bozza, S.; Camilloni, B.; Fusco-Moffa, I.; Monari, C.; De Robertis, E., et al. Is recurrence possible in coronavirus disease 2019 (COVID-19)? Case series and systematic review of literature. *Eur J Clin Microbiol Infect Dis* **2020**, 10.1007/s10096-020-04057-6, doi:10.1007/s10096-020-04057-6.

53. Gousseff, M.; Penot, P.; Gallay, L.; Batisse, D.; Benech, N.; Bouiller, K.; Collarino, R.; Conrad, A.; Slama, D.; Joseph, C., et al. Clinical recurrences of COVID-19 symptoms after recovery: Viral relapse, reinfection or inflammatory rebound? *J Infect* **2020**, *81*, 816-846, doi:10.1016/j.jinf.2020.06.073.
54. Li, Y.; Hu, Y.; Yu, Y.; Zhang, X.; Li, B.; Wu, J.; Li, J.; Wu, Y.; Xia, X.; Tang, H., et al. Positive result of Sars-Cov-2 in faeces and sputum from discharged patients with COVID-19 in Yiwu, China. *J Med Virol* **2020**, *92*, 1938-1947, doi:10.1002/jmv.25905.
55. Peng, J.; Wang, M.; Zhang, G.; Lu, E. Seven discharged patients turning positive again for SARS-CoV-2 on quantitative RT-PCR. *Am J Infect Control* **2020**, *48*, 725-726, doi:10.1016/j.ajic.2020.03.017.
56. Tao, W.; Wang, X.; Zhang, G.; Guo, M.; Ma, H.; Zhao, D.; Sun, Y.; He, J.; Liu, L.; Zhang, K., et al. Re-detectable positive SARS-CoV-2 RNA tests in patients who recovered from COVID-19 with intestinal infection. *Protein & cell* **2020**, 10.1007/s13238-020-00778-8, 1-6, doi:10.1007/s13238-020-00778-8.
57. Tomassini, S.; Kotecha, D.; Bird, P.W.; Folwell, A.; Biju, S.; Tang, J.W. Setting the criteria for SARS-CoV-2 reinfection: six possible cases. *J Infect* **2020**, 10.1016/j.jinf.2020.08.011, doi:10.1016/j.jinf.2020.08.011.
58. Zhang, B.; Liu, S.; Dong, Y.; Zhang, L.; Zhong, Q.; Zou, Y.; Zhang, S. Positive rectal swabs in young patients recovered from coronavirus disease 2019 (COVID-19). *J Infect* **2020**, *81*, e49-e52, doi:10.1016/j.jinf.2020.04.023.
59. Gagnier, J.J.; Kienle, G.; Altman, D.G.; Moher, D.; Sox, H.; Riley, D.; the, C.G. The CARE guidelines: consensus-based clinical case reporting guideline development. *J Med Case Rep* **2013**, *7*, 223, doi:10.1186/1752-1947-7-223.
